# Supplementary material for: Blocking RpoN reduces virulence of Pseudomonas aeruginosa isolated from cystic fibrosis patients and increases antibiotic sensitivity in a laboratory strain
Source: Sci Rep. 2019 Apr 30;9:6677. doi: 10.1038/s41598-019-43060-6 (PMC6491466; doi:10.1038/s41598-019-43060-6)
Supplement: Supplementary file 1 — Supplementary Figures [file 41598_2019_43060_MOESM1_ESM.pdf]

Supplemental Material:

Blocking RpoN reduces virulence of *Pseudomonas aeruginosa* isolated from cystic fibrosis patients and increases antibiotic sensitivity in a laboratory strain

Lloyd MG[1], Vossler JL[2], Nomura CT[3,4], Moffat JF[1*]

[1]Department of Microbiology and Immunology, SUNY Upstate Medical University, Syracuse, NY, USA

[2]Department of Clinical Laboratory Science, SUNY Upstate Medical University, Syracuse, NY, USA

[3]Department of Chemistry, SUNY College of Environmental Science and Forestry, Syracuse, NY, USA

[4]Center for Applied Microbiology, SUNY College of Environmental Science and Forestry, Syracuse, NY, USA

[*]Corresponding author

E-mail: moffatj@upstate.edu

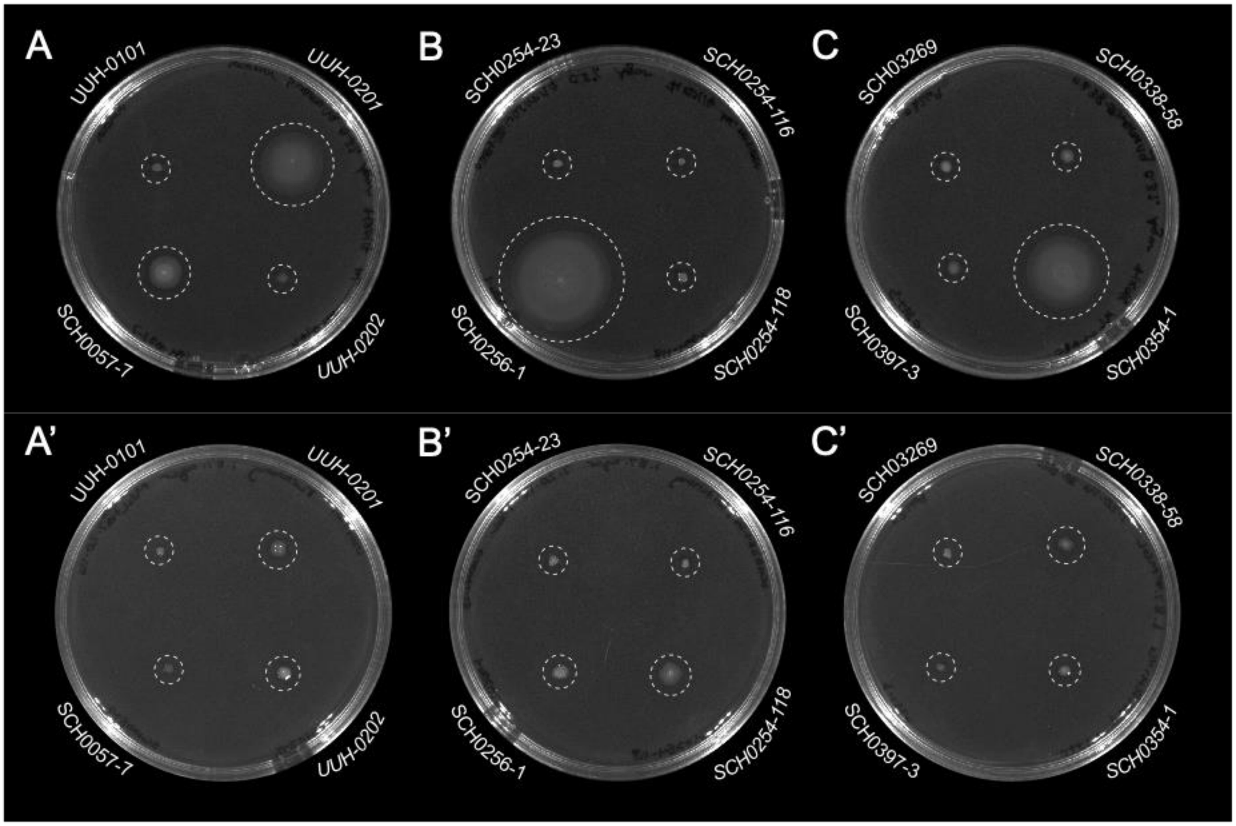

Supplemental Figure S1. The motility phenotypes of *P. aeruginosa* CF patient isolates are variable. Photograph of motility phenotypes for *P. aeruginosa* CF patient isolates. Strains include (clockwise from top left): (A and A'): UUH-0101, UUH-0201, UUH-0202, and SCH 0057-7; (B and B') SCH 0254-23, SCH 0254-116, SCH 0254-118, and SCH 0256-1; (C and C') SCH 03269, SCH 0338-58, SCH 0354-1, and SCH 0397-3. All assays were performed at 37°C for 24 h. (A-C) Colony diameter of swimming, or flagellar, motility assay conducted on soft (0.3%) agar. (A'-C') Colony diameter of twitching, or pili, motility assay conducted on semi-hard (1.3%) agar. White dotted circles indicate the location and size of each bacterial colony.

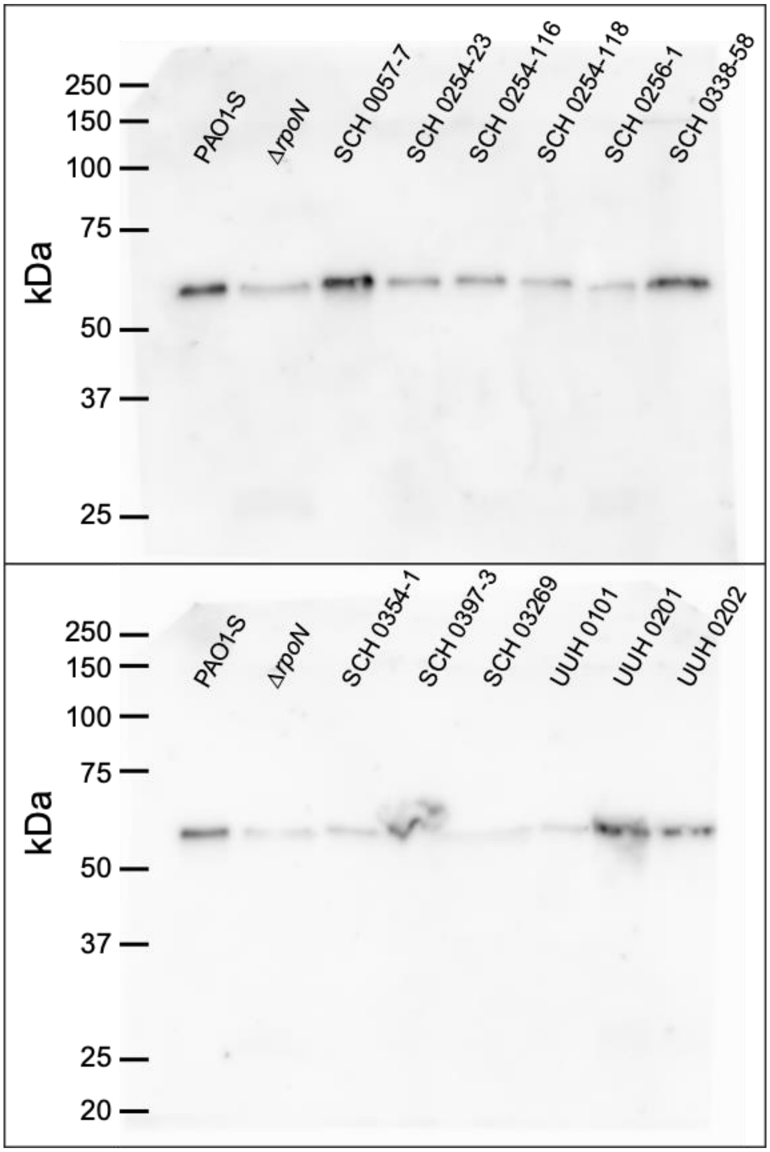

Supplemental Figure S2. Full Immunoblots of RpoN protein in *P. aeruginosa* isolates. Immunoblots of RpoN expression in *P. aeruginosa* CF patient isolates and laboratory strains PAO1-S and Δ*rpoN*. Immunoblots performed on 10% Mini-PROTEAN TGX Stain-Free gels (BioRad). Immunoblots were imaged in the same frame and under the same conditions. Immunoblots were imaged for 48.372 s (optimal auto-exposure).

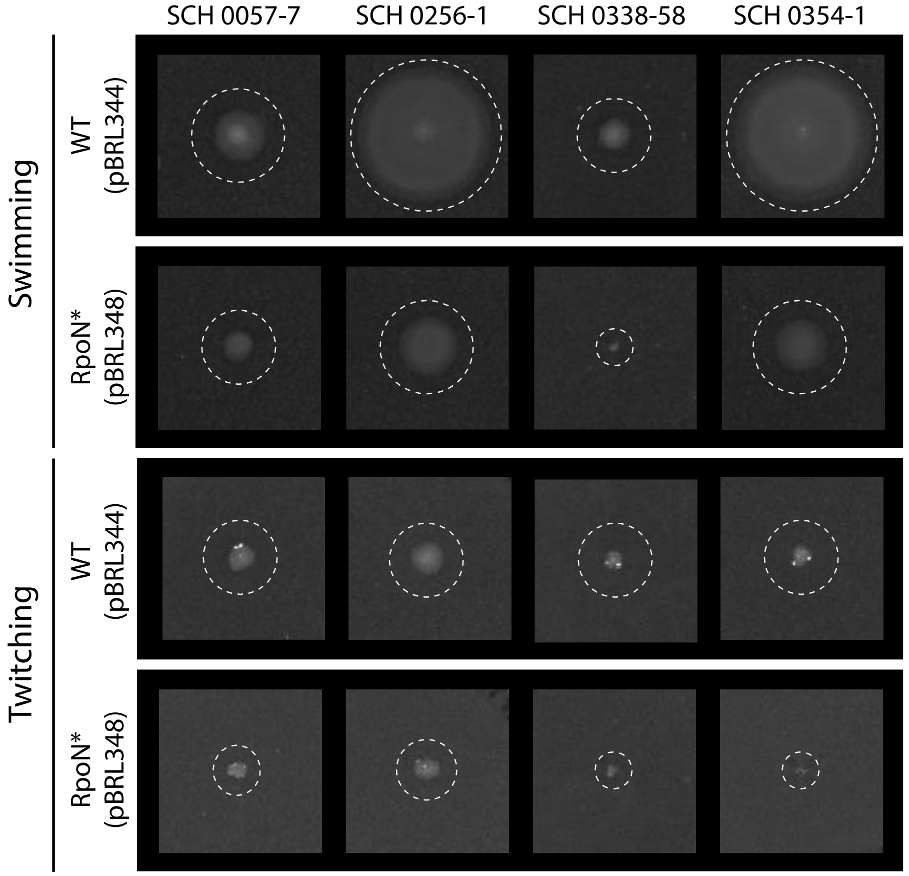

Supplemental Figure S3. RpoN* expression reduced motility in *P. aeruginosa* CF patient isolates. Photograph of motility phenotypes for transformable *P. aeruginosa* CF patient isolates including SCH 0057-7, SCH 0256-1, SCH 0338-58, and SCH 0354-1. Bacteria were transformed with empty vector (W.T., pBRL344), or with RpoN*-expression vector (pBRL348). The culture medium was supplemented with gentamicin (30 mg/L) and IPTG (1 mM), and assays were conducted at 37°C for 24 h. (First and Second Row) Colony diameter of swimming, or flagellar, motility assay conducted on soft (0.3%) agar in the absence (first row) or presence (second row) of RpoN*. (Third and Fourth Row) Colony diameter of twitching, or pili, motility assay conducted on semi-hard (1.3%) agar in the absence (third row) or presence (fourth row) of RpoN*. White dotted circles indicate the location and size of each bacterial colony.

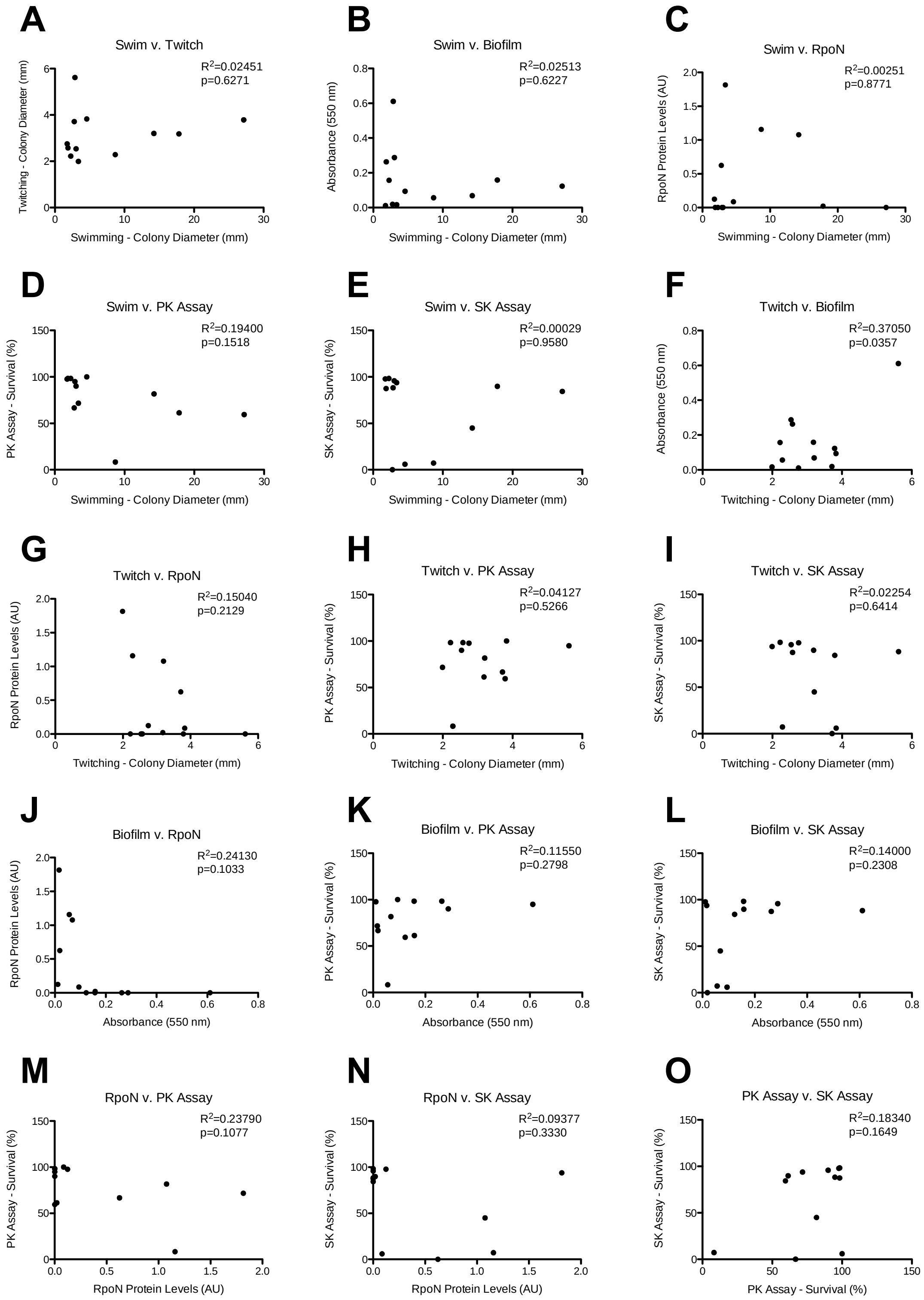

Supplemental Figure S4. Correlation analysis of *in vitro* and *in vivo* phenotypes for *P. aeruginosa* CF patient isolates. The results of the phenotyping assays presented in Figs. 1-3 were analyzed to determine the correlation between paired phenotypes. Correlation analyses

were performed using GraphPad Prism, and only twitching motility and biofilm formation were significantly correlated (panel F, p = 0.0357). (A) Swimming or flagella-associated motility and twitching or pili-associated motility. (B) Swimming motility and biofilm formation. (C) Swimming motility and RpoN protein levels. (D) Swimming motility and the paralytic killing infection model. (E) Swimming motility and the slow killing infection assay. (F) Twitching motility and biofilm formation. (G) Twitching motility and RpoN protein levels. (H) Twitching motility and the paralytic killing infection assay. (I) Twitching motility and the slow killing infection assay. (J) Biofilm formation and RpoN protein levels. (K) Biofilm formation and the paralytic killing infection assay. (L) Biofilm formation and the slow killing infection assay. (M) RpoN protein levels and the paralytic killing infection assay. (N) RpoN protein levels and the slow killing infection assay. (O) Paralytic killing and slow killing infection assays.
